# The Address before the American Institute

**Published:** 1882-08

**Authors:** 


The Address Before the American Institute. By the President, Dr. Breyfogle. Published by the Institute. 1882.

Through the courtesy of Dr. J. C. Burgher, we have received a copy of Dr. Breyfogle's address. There are many subjects, relating to Homœopathy, which might have been discussed with great advantage, but Dr. Breyfogle evidently "knew his audience," and hence skipped them! His suggestions as to the necessity of having a bureau of pharmacy are timely and necessary. The proposition to "limit the dose" by *resolution* is absurd; the doctor must be a great joker! Perhaps, in the address next year, the Institute will be urged to "resolve" that water shall not run down hill, or the sun be requested to cease shining!

